# Remodeling of Hepatocyte Mitochondrial Metabolism and *De Novo* Lipogenesis During the Embryonic-to-Neonatal Transition in Chickens

**DOI:** 10.3389/fphys.2022.870451

**Published:** 2022-04-21

**Authors:** Chaitra Surugihalli, Linda S. Farley, Ronique C. Beckford, Boonyarit Kamkrathok, Hsiao-Ching Liu, Vaishna Muralidaran, Kruti Patel, Tom E. Porter, Nishanth E. Sunny

**Affiliations:** ^1^ Department of Animal and Avian Sciences, University of Maryland, College Park, MD, United States; ^2^ Institute of Research and Development, Suranaree University of Technology, Nakhon Ratchasima, Thailand; ^3^ Department of Animal Science, NC State University, Raleigh, NC, United States

**Keywords:** chicken, liver metabolism, mitochondria, lipid oxidation, *de novo* lipogenesis, metabolomics

## Abstract

Embryonic-to-neonatal development in chicken is characterized by high rates of lipid oxidation in the late-term embryonic liver and high rates of *de novo* lipogenesis in the neonatal liver. This rapid remodeling of hepatic mitochondrial and cytoplasmic networks occurs without symptoms of hepatocellular stress. Our objective was to characterize the metabolic phenotype of the embryonic and neonatal liver and explore whether these metabolic signatures are preserved in primary cultured hepatocytes. Plasma and liver metabolites were profiled using mass spectrometry based metabolomics on embryonic day 18 (ed18) and neonatal day 3 (nd3). Hepatocytes from ed18 and nd3 were isolated and cultured, and treated with insulin, glucagon, growth hormone and corticosterone to define hormonal responsiveness and determine their impacts on mitochondrial metabolism and lipogenesis. Metabolic profiling illustrated the clear transition from the embryonic liver relying on lipid oxidation to the neonatal liver upregulating *de novo* lipogenesis. This metabolic phenotype was conserved in the isolated hepatocytes from the embryos and the neonates. Cultured hepatocytes from the neonatal liver also maintained a robust response to insulin and glucagon, as evidenced by their contradictory effects on lipid oxidation and lipogenesis. In summary, primary hepatocytes from the embryonic and neonatal chicken could be a valuable tool to investigate mechanisms regulating hepatic mitochondrial metabolism and *de novo* lipogenesis.

## Introduction

The United States has one of the world’s largest poultry production systems, producing over 9.2 billion broilers and 9.3 billion dozen eggs in 2020 (USDA, 2018). Together, there is a burden on the industry and the farmers to meet the expected demand for 128 million tons of poultry meat by 2022 (Davis et al., 2013). In this regard, early hatchling mortality (which ranges between 0.5 and 2%) during first 10 days after hatch is one of the major factors affecting broiler performance, quality and the economics of poultry industry (Noble et al., 1986; Wilson, 1991; Xin et al., 1994; Peebles et al., 2004; Pedroso et al., 2005; Yassin et al., 2009). The late embryonic and early neonatal development period in chicken is associated with dynamic developmental and metabolic changes in order to facilitate the rapid and healthy transition of the embryo to a hatchling, while simultaneously getting accustomed to the new carbohydrate rich dietary environment (Hicks et al., 2017; Surugihalli et al., 2019). Considering the relevance of this embryonic-to-neonatal period towards the overall health of the chicken, identifying nutritional and metabolic factors influencing this transition and in turn reducing mortality rates after hatch, is of significant interest to the poultry production systems.

The late term chicken embryo extensively relies on the yolk (>45% lipids) to derive over 90% of its energy, while the neonatal chicken has to rapidly transition to a carbohydrate rich starter diet (Hicks et al., 2017; Surugihalli et al., 2019). Thus, the embryonic liver in the chicken is programed to utilize free fatty acids through upregulation of the lipid oxidation networks. However, once exposed to a carbohydrate rich environment, the neonatal chicken liver undergoes a rapid metabolic switch from lipid utilization/oxidation to *de novo* lipogenesis from carbohydrates (Hicks et al., 2017). Such extremes of high rates of lipid oxidation and high rates of *de novo* lipogenesis, as evident during the embryonic-to-neonatal transition in chicken, is also a central feature of the mitochondrial co-morbidities in the liver of mice and humans with non-alcoholic fatty liver disease (NAFLD) (Sunny et al., 2011; Lambert et al., 2014; Patterson et al., 2016; Sunny et al., 2017). Such metabolic milieu could also be a contributing factor to the onset of fatty liver hemorrhagic syndrome (FLHS) in older layer and broiler flocks (Walzem et al., 1993; Lee et al., 2010; Pan et al., 2012; Trott et al., 2013; Liu et al., 2016). Furthermore, during these co-morbidities, high rates of lipid oxidation and high rates of *de novo* lipogenesis in the liver co-exist with inflammation and indices of hepatocellular stress (Nakamura et al., 2009; Satapati et al., 2016; Sunny et al., 2017). Interestingly, despite the lipid overburden associated with the high rates of lipid oxidation and high rates of *de novo* lipogenesis, the embryonic-to-neonatal transition occurs without any major evidence of cellular stress and inflammation in the liver (Surugihalli et al., 2019). Thus, while the existence of this dynamic “metabolic switch” during embryonic-to-neonatal transition is well known (Hicks et al., 2017; Cogburn et al., 2018; Surugihalli et al., 2019), the nutritional, metabolic and molecular factors mediating this transition in chicken remain unclear.

Mitochondrial oxidative networks in the liver, which include β-oxidation, ketogenesis, tricarboxylic acid (TCA) cycle and electron transport chain, adapt and remodel in response to different nutrient and hormonal cues under normal physiological states and during stages of insulin resistance (Satapati et al., 2012; Koliaki et al., 2015; Sunny et al., 2017). Indeed, dysfunctional mitochondrial metabolism is a central feature in rodent and human models of metabolic diseases such as insulin resistance, type-2 diabetes (T2DM) and fatty liver disease (Jelenik et al., 2017; Sunny et al., 2017) and could be a contributing factor to FLHS onset in older layer and broiler flocks (Walzem et al., 1993; Trott et al., 2013). Remodeling of mitochondrial metabolism during insulin resistance and fatty liver disease is characterized by selective activation of certain mitochondrial and cytoplasmic networks, which include the TCA cycle and *de novo* lipogenesis, respectively (Sunny et al., 2011; Lambert et al., 2014; Patterson et al., 2016; Surugihalli et al., 2019). The chronic induction of these networks is thought to aggravate hepatic stress and inflammation during fatty liver disease (Sunny et al., 2011; Satapati et al., 2016). The metabolic milieu in the liver during the embryonic-to-neonatal transition in the chicken allows us to investigate the relevance of the remodeling of mitochondrial networks and lipogenesis towards preserving hepatocyte function.

Our primary objective was to characterize the metabolic profiles of the plasma and liver in chicken embryos and neonatal chicks, in order to demonstrate the dramatic transition from lipid utilization in the embryos to lipid synthesis in the neonates. We further tested whether isolated primary hepatocytes from embryos and neonatal chicks accurately reflect the metabolic adaptation from embryonic-to-post hatch, in order to validate its utility as an *in vitro* system to probe factors regulating mitochondrial function and *de novo* lipogenesis.

## Materials and Methods

### Experimental Plan

Eggs (64 g ± 0.6 standard error of means; SEM) were obtained from Perdue Farms Inc. (Salisbury, MD) from a broiler flock (Ross 708), and were incubated at 37°C and 45% relative humidity. On the day of hatch (day 21), neonatal chicken were transferred to floor pens maintained at 37°C and were provided a starter diet (Diet S-G 5065; ASAP Feed and Bedding, Quakertown, PA) *ad libitum*. The day-18 embryos (ed18) were sacrificed by decapitation, and the neonatal day-3 chicken (nd3) were decapitated following cervical dislocation. Plasma and tissue samples were collected from mixed-sex birds and frozen at −80°C for future analyses. Livers from a subset of day-18 embryos and day-3 neonatal chicken were utilized for the isolation of primary hepatocytes as detailed below. All the experiments were conducted in accordance with Institutional Animal Care and Use Committee protocols approved at the University of Maryland, College Park. All experiments were performed in accordance with relevant guidelines and regulations described by our institutional animal care and use committee. The methods and protocols in this study is reported in accordance with ARRIVE guidelines.

### Isolation and Culturing of Primary Hepatocytes

In a separate set of experiments, livers from embryonic day-18 and neonatal day-3 broiler chickens were utilized of isolation of the hepatocytes. Following euthanasia by cervical dislocation and decapitation, the birds were cut open to expose the heart and the liver. The left ventricle was infused with a perfusion media (10 ml basic SMEM and 20 µl 0.5 M EGTA) and the hepatic vein was severed. Following this, 20 ml SMEM media with collagenase and 0.1% bovine serum albumin (BSA) was infused. This media consisted of 500 ml SMEM (Minimal Essential media for Suspension cultures, Gibco); 57 ml 10X HBSS (Hank’s Buffered Salt Solution, Gibco); 5.7 ml PenStrep (10,000 U/ml penicillin, 10,000 U/ml streptomycin) and 7.6 ml of BSA with Collagenase (0.12%) and 4 mM calcium. The perfused liver was collected and minced into small pieces in SMEM media with collagenase in a petri dish and incubated for 10 min at 37°C in a CO_2_ incubator. The minced liver pieces were then filtered through a 70 µM filter into a 50 ml centrifuge tube and an additional 20 ml of SMEM media with EGTA was added to it. Later, 10 ml of 50% percol (in SMEM media with EGTA) was added and centrifuged. The cells were then pelleted by centrifuging and washed to remove any dead cells and debris. This was repeated three times. One experimental unit consisted of pooled cells from 2–3 livers, in order to obtain optimal hepatocyte cell counts for seeding the plates. Cells were then re-suspended in Williams’ media [500 ml Williams’ E media with 5.2 ml 1 mM HEPES (10 mM final concentration), 5.2 ml 100X L-glutamine (1X final concentration), 5.2 ml PenStrep and 7 ml 7.5% BSA (0.1% final concentration)] at 10^6^ cells/ml. The cells were plated in 6-well culture plates pre-coated with 0.1% gelatin, and the hormonal treatments were added after 3 h. The cells were either treated with the basal media, corticosterone, growth hormone, glucagon or insulin for 24-h. All hormonal treatments were added at a final concentration of 10^−9^ M. Following 24-h of treatment, the cells were washed twice with ice-cold PBS and harvested using 1X RIPA and frozen at −80°C for further metabolic analysis. All the media were filtered and gassed for 30 s with 95% oxygen and 5% carbon dioxide. Following overnight culture, a set of basal hepatocytes from ed18 and nd3 were treated with 0, 25, and 100 nM insulin for 10 min, to determine the insulin signaling response of the hepatocytes. Following 10 min of insulin incubation, the cells were washed with cold phosphate buffered saline two times, collected and frozen in a lysis buffer for western blot analysis.

### Analysis of TCA Cycle Organic Acids and Amino Acids by Gas Chromatography-Mass Spectrometry

Serum (25 µl) collected from ed18 and nd3 broiler birds were spiked with an equal volume of stable isotope-labelled internal standards and were deproteinized with 700 µl of 70% acetonitrile. The samples were centrifuged at 13,500 rpm for 15 min at 4°C, and the supernatant was transferred to a 1 ml v-vial and dried under a stream of nitrogen gas. The metabolites were then converted to their oximes with the addition of 20 µl of 2% methoxamine hydrochloride in pyridine (W/V) and microwaving at 350 W for 90 s. The samples were then derivatized with TBDMS (Tert-butyldimethylsilyl) at 90°C for 1 h. The metabolites were separated on a HP-5MS UI column (30 m × 0.25 mm × 0.25 μm; Agilent, CA, United States) and the ion fragments determined by single ion monitoring (SIM) under electron ionization mode using a GC-MS (5973N, Mass Selective Detector coupled to a 6890 Series GC System, Agilent, CA, United States). Metabolite concentrations were determined in relation to their respective stable isotope-labelled internal standard. For primary hepatocytes, the cells were collected in 1X RIPA and were deproteinized with 700 µl of 70% acetonitrile. The samples were spiked with a known volume of stable isotope-labeled organic acid and amino acid internal standards and sonicated for 10-min to extract the cellular contents. This extract was processed similar to the serum samples for GC-MS analysis.

### Analysis of Triglyceride-Fatty Acids in Primary Hepatocytes

GC-MS analysis of the fatty acid methyl esters was performed as previously described (Surugihalli et al., 2019). Briefly, hepatocytes were collected using 1X RIPA and lipids were Folch extracted with 750 μl chloroform: methanol (2:1) after the addition of a mixed U^13^C fatty acid internal standard (Cambridge isotopes, MA, United States). The lipid layer was dried and saponified with 0.5 N methanolic NaOH for 30 min at 50°C. The samples were treated with 1 ml of 2% methanolic sulphuric acid and incubated at 50°C for 2-h to form fatty acid methyl esters (FAMEs). The FAMEs were then extracted with hexane, dried and re-suspended in 50–100 μl of hexane for GC-MS analysis. The FAMEs were separated on a VF 23 ms column (30 m × 0.25 mm × 0.25 μm; Agilent, CA, United States), followed by fragmentation under electrical ionization on a 5973N-Mass Selective Detector, 6890-Series GC, (Agilent, CA, United States). Concentrations of individual FAMEs were determined relative to their respective stable isotope-labeled internal standard.

### Western Blot Analysis

Primary hepatocytes treated under basal and insulin-stimulated conditions were collected in 100 µl of 1x RIPA containing protease and phosphatase inhibitor and incubated for 1 h on ice to extract proteins. The cells were then centrifuged at 13,500 rpm for 15 min at 4°C. The protein content of the supernatant was determined using BCA protein assay (Thermo Fischer Scientific. Waltham, MA, United States). Proteins (10 µg) were separated on 8% tris bolt gels (Invitrogen, Carlsbad, CA, United States), and were transferred to a nitrocellulose membrane and incubated with primary antibodies Akt, pAkt and GAPDH (Cell Signaling Technology Inc., Danvers, MA, United States) to evaluate the response of the primary hepatocytes to insulin treatment.

### Global Metabolomics and Lipidomics of Liver Tissue

Liver tissue from a previously published set of studies (Hicks et al., 2017) were utilized for global metabolomics and lipidomics. In this previously published study, eggs from specific pathogen-free (SPF) leghorn chickens (layers) were obtained from Charles River Laboratories (Wilmington, MA, United States) and incubated at 37.5°C and 60% relative humidity with rotation every hour. Livers from embryonic day 18 (ed18; *n* = 9) and neonatal day 3 (nd3: *n* = 9) chickens (mixed-sex) were utilized for global metabolomics and lipidomics. For the metabolomic and lipidomic analyses, the liver tissues were pre-normalized for mass spectrometry at a protein concentration of 500 μg/ml. Liver samples (25 mg) underwent Folch extraction and the aqueous and lipid layers were was dried and reconstituted for LC-MS/MS based metabolomics and lipidomics, respectively. Global metabolomics and lipidomics was performed on a Thermo Q-Exactive Oribtrap mass spectrometer with Dionex UHPLC and autosampler. All samples were analyzed in positive and negative heated electrospray ionization with a mass resolution of 35,000 at m/z 200 as separate injections. Separation was achieved on an ACE 18-pfp 100 × 2.1 mm, 2 μm column for polar metabolites. Separation was achieved on Acquity BEH C18 1.7 μm, 100 mm × 2.1 mm column for lipid metabolites.

### Gene Expression Profiles From RNA Sequencing

RNA sequencing data from SPF leghorn chicken layer livers, a previously published data set (Hicks et al., 2017), were utilized to evaluate changes in expression levels of specific genes involved in mitochondrial metabolism and lipogenesis. In brief, total RNA was isolated and purified using Tri-Reagent (Sigma) per manufacturer’s protocol from the liver tissue of e18 and nd3 chicken (*n* = 4 per group). Small RNA libraries (1 µg/each library) were developed at using TruSeq Small RNA sample preparation kit (Illumina) per manufacturer’s protocol. The diluted library (10 nM) from each bird was pooled and sequenced with Illumina Genome Analyzer IIx (GAIIx) (NCSU Genomic Sciences Laboratory). The mRNA libraries were generated using TruSeq RNA library preparation kit v2 (Illumina) and barcode indices following the manufacturer’s instructions and were assessed using high sensitivity DNA chip on a Agilent Technologies 2100 Bioanalyzer. Further a 50 bp single end of each library was sequenced at DHMRI (Kannapolis, NC) using an Illumina HiSeq 2500. The sequencing data was processed and analyzed CLC genomics workbench (Qiagen).

### Gene Expression Analysis Using RT-qPCR

Gene expression analysis performed as previously described (Beckford et al., 2020). Total RNA from primary hepatocytes, derived from broiler chicken livers, was isolated using the RNeasy mini kit (Qiagen, Hilden, Germany) following the manufacturer’s protocol. Before elution of RNA, an on-column deoxyribonuclease digestion was done to remove genomic DNA. Total RNA was quantified using Quant-iT RiboGreen RNA Quantification reagent (Invitrogen, Carlsbad, CA, United States). Following which cDNA was prepared from1 μg of total RNA in 20 μl reactions using M-MLV RT kit (New England Biolabs, Ipswich, MA) following the manufacturer’s instructions. Quantitative real-time PCR was performed using 20 ng of cDNA, 10 µM of each primer, and 7.5 μl of 2X master mix [PCR buffer (50 mM KCl, 10 mM Tris–HCl, 0.1% triton-X-100), 0.12 U/μl Taq Polymerase, 0.2 μM dNTPs, 40 nM fluorescein (Invitrogen, Waltham, MA, United States), and SYBR Green I Nucleic Acid Gel Stain diluted 1:10,000 (Invitrogen, Waltham, MA, United States), and 4.3 μl of water. Samples were run in duplicate and PCR was performed for 40 cycles under the following conditions: 95°C for 15 s, 60°C for 30 s, and then 72°C for 30 s. Target genes were normalized to the expression of *GAPDH* in the same samples and then expressed relative to the basal treatment (untreated cells). Primers (Supplementary Table 1) for real time quantitative PCR. The genes were designed using Primer Express 2.0 (Applied Biosystems, Waltham, MA, United States) and validated according to the procedures previously described (Ellestad et al., 2011).

### Statistical Analysis

All the data reported are presented as means ± standard error of means (SEM). Results were analyzed using unpaired *t*-tests for comparisons between e18 and nd3. The responses to hormonal treatments were analyzed using one-way ANOVA followed by Tukey’s pair-wise means comparisons. Means were considered significantly different at *p* ≤ 0.05. All the statistical analysis were conducted and the graphs were plotted utilizing Prism 7 (GraphPad software Inc., San Diego, CA, United States).

## Results

### Alterations in Plasma Metabolites Highlight the Major Metabolic Shift During Embryonic-to-Neonatal Development

**FIGURE 1:**
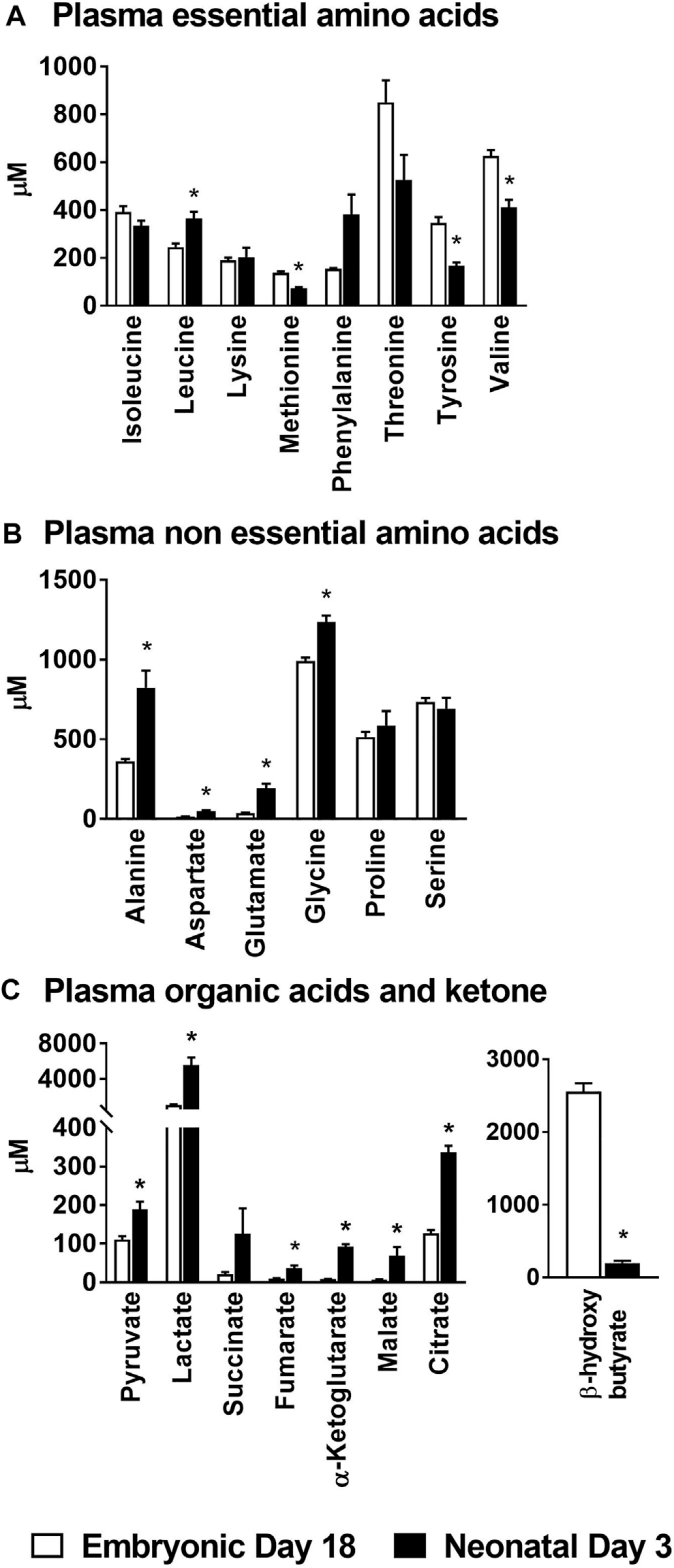
Changes in plasma amino acids, organic acids and β-hydroxybutyrate during embryonic-to-neonatal development. Plasma concentrations of **(A)** Essential amino acids; **(B)** Non-essential amino acids; **(C)** Organic acid intermediates of the TCA cycle and β-hydroxybutyrate in broiler embryos and neonates. All the values are represented as means ± SEM with *n* = 6–9 birds/group. Results were considered significant at *p* ≤ 0.05 (*) following a Student *t*-test between embryonic day 18 and neonatal day 3.

The circulating concentration (µM ± SEM) of the essential amino acid leucine (ed18: 246 ± 15 vs. nd3: 365 ± 28) was significantly higher (*p* ≤ 0.05) in the neonatal chicks; while those of methionine (ed18: 138 ± 6 vs. nd3: 73 ± 5), tyrosine (ed18: 347 ± 24 vs. nd3: 166 ± 15) and valine (ed18: 626 ± 25 vs. nd3: 411 ± 32) were significantly lower (*p* ≤ 0.05) than their embryonic counterparts (Figure 1A). Plasma non-essential amino acids alanine (ed18: 363 ± 13; nd3: 825 ± 107), aspartate (ed18: 15 ± 2; nd3: 49 ± 7), glutamate (ed18: 38 ± 2; nd3: 194 ± 27), and glycine (ed18: 991 ± 22; nd3: 1,236 ± 42), were significantly higher (*p* ≤ 0.05) in the neonatal chicken compared to their embryonic counterparts (Figure 1B). There was also a parallel increase in plasma organic acids pyruvate (ed18: 111 ± 8 vs. nd3: 189 ± 20), lactate (ed18: 1,042 ± 71 vs. nd3: 5,573 ± 831), and all the mitochondrial TCA cycle intermediates, succinate (ed18: 21 ± 5 vs. nd3: 126 ± 65), fumarate (ed18: 9 ± 1 vs. nd3: 36 ± 7), α-ketoglutarate (ed18: 8 ± 1 vs. nd3: 92 ± 7), malate (ed18: 7 ± 1 vs. nd3: 68 ± 23), and citrate (ed18: 127 ± 9 vs. nd3: 336 ± 17), in the neonatal chicken (*p* ≤ 0.05, Figure 1C). On the contrary, there was a dramatic decrease in the plasma β-hydroxybutyrate levels in the neonatal chicks compared to their embryonic counterparts (ed18: 2,556 ± 116 vs. nd3: 200 ± 31; *p* ≤ 0.05) (Figure 1C). These observed differences in circulating levels of amino acids, organic acids and β-hydroxybutyrate point to significant remodeling of energy metabolism between embryonic and neonatal stages in chicken.

### Remodeling of Hepatic Metabolism during Embryonic-to-Neonatal Development

**FIGURE 2:**
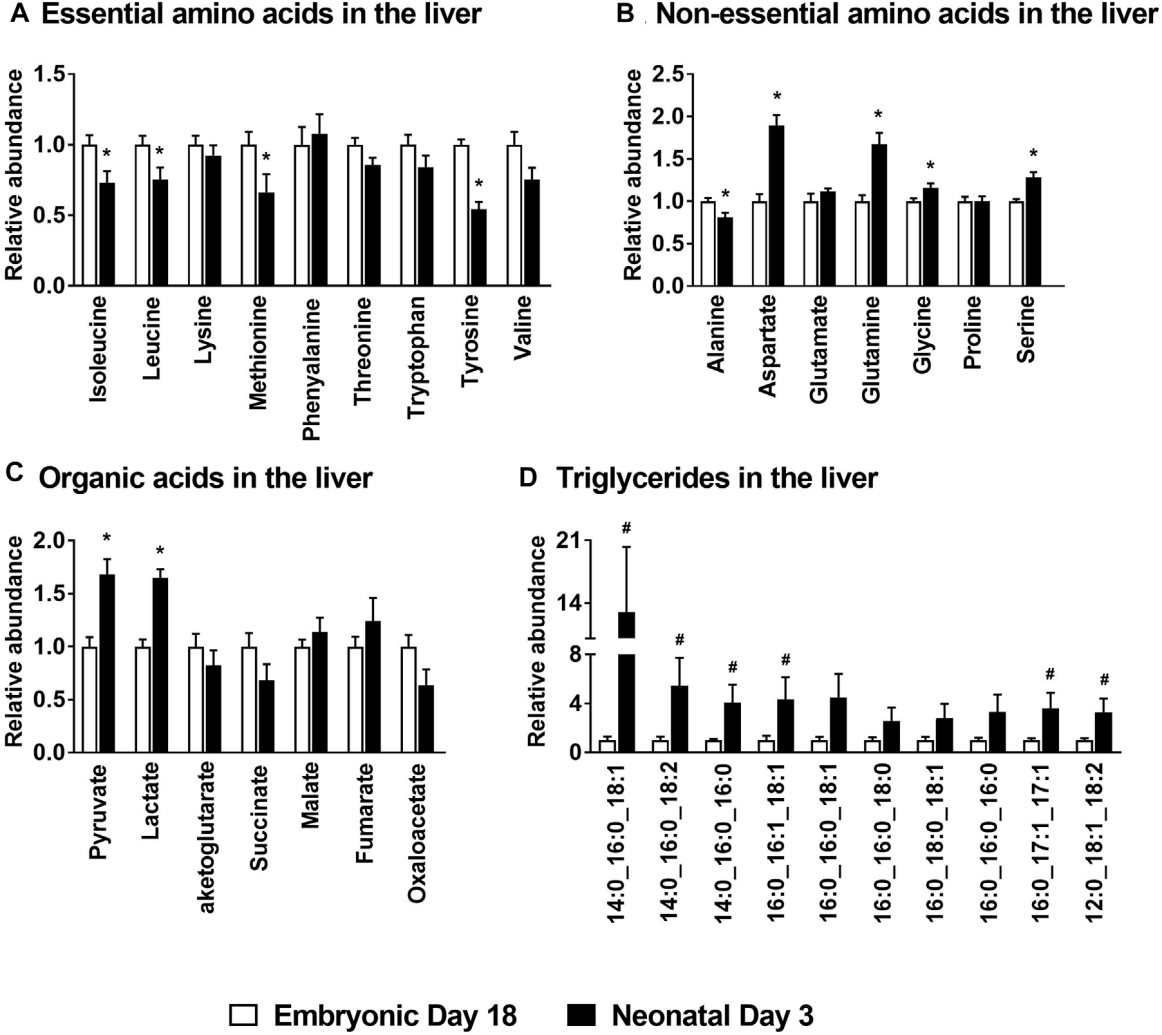
Metabolite profile of the embryonic and neonatal chicken liver. Changes in neonatal liver metabolites are presented relative to those of embryonic day 18. Profiles of **(A)** Essential amino acids; **(B)** Non-essential amino acids; **(C)** Organic acids associated with mitochondrial TCA cycle and **(D)** Hepatic triglycerides in embryos and neonates from leghorn layer flock. All the values are represented as means ± SEM with *n* = 6–9 birds/group. Results were considered significant at *p* ≤ 0.05 (*) or a trend at *p* ≤ 0.1 (^**#**^) following a Student *t*-test between embryonic day 18 and neonatal day 3.

There was a significant decrease (*p* ≤ 0.05) in the levels of the essential amino acids isoleucine, leucine, methionine and tyrosine in the liver of the neonatal chicks compared to their embryonic counterparts (Figure 2A). While the levels of the non-essential amino acid alanine was significantly lower (*p* ≤ 0.05) in neonatal chicks, aspartate, glutamine, glycine and serine were significantly higher (*p* ≤ 0.05) in neonatal chicken liver compared to the embryonic liver (Figure 2B). Pyruvate and lactate levels were significantly higher (*p* ≤ 0.05) in the neonatal liver whereas the mitochondrial TCA cycle intermediates remained unchanged between the two groups (Figure 2C). Concurrently, profiling of various triglycerides in the liver demonstrated a 2–10 fold increase (*p* ≤ 0.1) in their abundances in the neonatal chicken liver compared to their embryonic counterparts (Figure 2D). Considering the dramatic differences in the total liver weight and the total protein content of the liver between the ed18 and nd3 livers (Surugihalli et al., 2019), tissue samples were pre-normalized based on a known amount of liver protein, before liquid chromatography-mass spectrometry (LC-MS/MS) analysis, for the analysis and comparison of triglycerides between the two groups.

### Hepatic Gene Expression Profiles Clearly Illustrate the Metabolic Shift From Lipid Oxidation in the Embryonic Liver to New Lipid Synthesis in the Neonatal Liver

**FIGURE 3:**
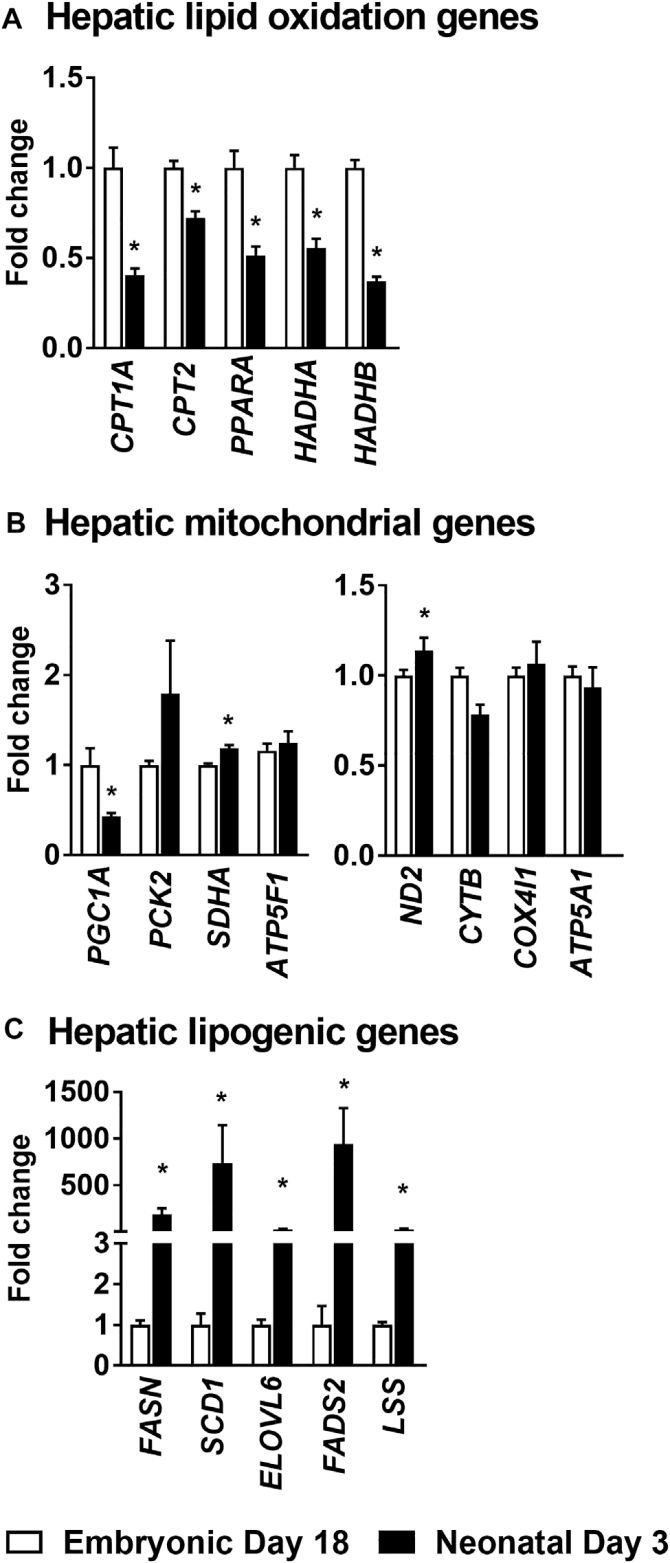
Changes in hepatic gene expression profiles during embryonic-to-neonatal development. The fold changes in the expression profiles of genes involved in **(A)** Lipid oxidation; **(B)** Mitochondrial metabolism and **(C)** Lipogenesis, between the embryonic and neonatal liver. RNA sequencing data from a previously published data set, from a leghorn layer background, were utilized for these analysis (Hicks et al., 2017). All the values are represented as means ± SEM with *n* = 4 birds/group. Results were considered significant at *p* ≤ 0.05 (*) following a Student *t*-test between embryonic day 18 and neonatal day 3. ***CPT1A***, Carnitine palmitoyl transferase 1a. ***CPT2***, Carnitine palmitoyl transferase 2. ***PPARA***, Peroxisome proliferator activator receptor 1a. ***HADHA***, Hydroxyacyl-CoA dehydrogenase subunit A. ***PGC1A***, Peroxisome proliferator-activated receptor gamma coactivator 1-alpha. ***PCK2***, Phosphoenolpyruvate carboxykinase 2. ***SDHA***, Succinate dehydrogenase flavoprotein subunit A. ***ATP5F1***, ATP synthase F (0) complex subunit B1. ***ND2***, NADH dehydrogenase subunit 2. ***CYTB***, Cytochorome b. ***COX4I1***, Cytochrome c oxidase subunit 4. ***ATP5A1***, ATP synthase alpha subunit. ***FASN***, Fatty acid synthase. ***SCD1***, Steroyl-CoenzymeA desaturase1. ***ELOVL6***, Elongation of very long chain fatty acids protein. ***FADS2***, Fatty Acid Desaturase 2. ***LSS***, Lanosterol synthase.

The lipid oxidation gene carnitine palmitoyl transferase 1a (*CPT1A*), which is involved in the formation of fatty acyl-carnitines and facilitate the movement of fatty acids in to the mitochondria for β-oxidation was downregulated 2–3 fold (*p* ≤ 0.05) in the neonatal chicken liver (Figure 3A). Similarly, Carnitine palmitoyl transferase 2 (*CPT2*), which converts fatty acyl-carnitine to fatty acyl-CoA and carnitine, for the breakdown of free fatty acids through β-oxidation, was also suppressed ∼2-fold (*p* ≤ 0.05) in the neonatal liver (Figure 3A). Furthermore, the gene expression of peroxisome proliferator activator receptor 1a (*PPARA*), a transcription factor which is a master regulator of lipid oxidation and hydroxyacyl-CoA dehydrogenase subunit A (*HADHA*) an enzyme involved in mitochondrial β-oxidation were also downregulated ∼2–3 fold (*p* ≤ 0.05) post-hatch (Figure 3A). The decrease in mitochondrial fat oxidation was associated with higher expression of certain mitochondrial complex genes such as succinate dehydrogenase subunit a (*SDHA*) and NADH dehydrogenase (*ND2*) in neonatal chicken liver (Figure 3B). However, hepatic gene expression of *PGC1A*, a gene that codes for mitochondrial biogenesis was lower in the neonatal liver (*p* ≤ 0.05; Figure 3B). The lower expression of the lipid oxidation genes in the liver of the neonatal chicken was accompanied by a ∼100–200 fold increase in the expression of genes involved in lipogenesis (Figure 3C) including fatty acid synthase (*FASN*), steroyl-Coenzyme A destaurase1 (*SCD1*), elongation of long-chain fatty acids family member 6 (*ELOVL6*), fatty acid desaturase 2 (*FADS2*) and lanosterol synthase (*LSS*). Taken together, these data highlight the metabolic transition from high rates of lipid oxidation and free fatty acid utilization by the embryonic liver, to high rates of *de novo* lipogenesis by the neonatal liver.

### Isolated Primary Hepatocytes From the Neonatal Chicken Accumulated More Lipids and Displayed a Robust Response to Insulin Treatment

**FIGURE 4:**
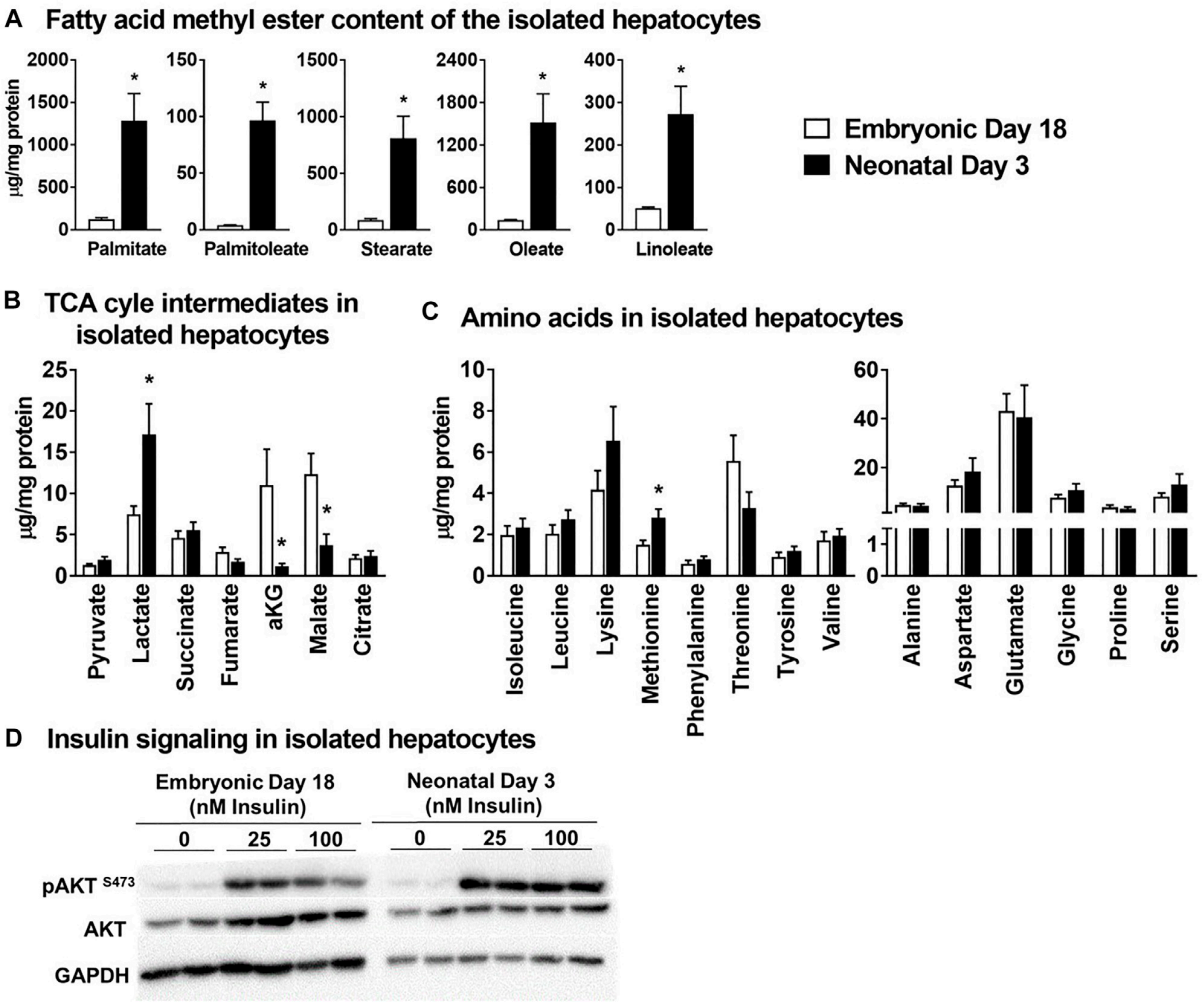
Primary isolated hepatocytes from broiler chicken embryos and neonates - a metabolic model to investigate mitochondrial metabolism and lipid accumulation. All metabolite concentrations were normalized to the cell protein content of the isolated hepatocytes. **(A)** Concentrations of triglyceride derived fatty acid methyl esters in the hepatocytes, **(B)** Levels of TCA cycle intermediates in the isolated hepatocytes **(C)** levels of essential and non-essential amino acids in the isolated hepatocytes and, **(D)** western blot analysis of pAKT at Ser-473 as an index of insulin signaling. All the values are represented as means ± SEM with *n* = 4–6 independent replicates/group. Each replicate consisted of pooled hepatocytes isolated from 2–3 birds. Results were considered significant at *p* ≤ 0.05 (*) following a Student *t*-test between embryonic day 18 and neonatal day 3. AKT, Protein kinase B.

Triglycerides were isolated from primary hepatocytes and converted to their fatty acid methyl esters for gas chromatography-mass spectrometry (GC-MS) analysis. The concentrations (µg/mg ± SEM) of triglyceride-fatty acid methyl ester of palmitate (ed18: 123 ± 21 vs. nd3: 1,284 ± 320), palmitoleate (ed18: 4.0 ± 0.40 vs. nd3:; 96.7 ± 15.9), stearate (ed18: 88 ± 14 vs. nd3: 809 ± 196), oleate (ed18: 139 ± 6 vs. nd3: 1,519 ± 403) and linoleate (ed18: 51 ± 3 vs. nd3: 272 ± 66) were significantly higher (*p* ≤ 0.05) in the hepatocytes isolated from neonatal day 3 chicks (Figure 4A). For the organic acid intermediates of the TCA cycle, the lactate content (µg/mg ± SEM; ed18: 7.45 ± 1.02 vs. nd3: 17.15 ± 3.73) was significantly higher (*p* ≤ 0.05) in hepatocytes from neonatal chicks (Figure 4B), while fumarate, α-ketoglutarate and malate were significantly lower (*p* ≤ 0.05) in the neonatal hepatocytes (Figure 4B). The concentration of all amino acids remained similar between groups (Figure 4C), except for methionine which was significantly higher (*p* ≤ 0.05) in the neonatal chick hepatocytes (ed18: 1.51 ± 2.52 vs. nd3: 2.83 ± 0.41) (Figure 4C). Further, there was a robust response of the hepatocytes from both the embryonic and neonatal groups to insulin treatment, as detected by the phosphorylation of AKT (protein kinase B) at serine 473 (Figure 4D). The accumulation of lipids by the neonatal primary hepatocytes and their response to insulin were similar to those observed in an *in vivo* embryonic-to-neonatal transition setting. Thus, the primary hepatocyte model system could have utility towards exploring factors regulating lipid accumulation, mitochondrial metabolism and insulin signaling.

### Impact of the Hormonal stimuli on Hepatocyte Mitochondrial Lipid Metabolism and Lipogenesis

**FIGURE 5:**
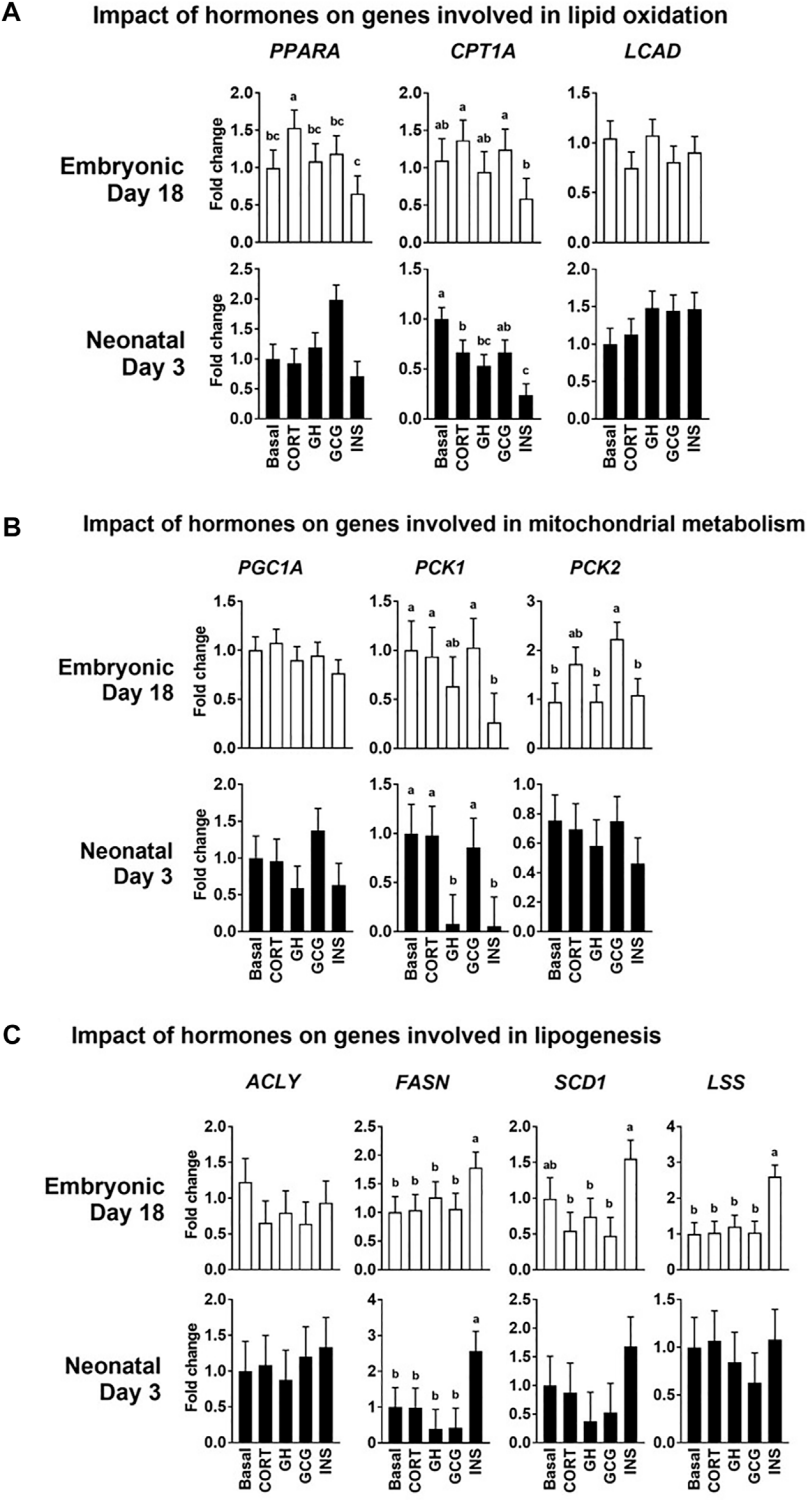
Impact of the hormonal stimuli on hepatocyte metabolism. Quantitative PCR analysis was performed to determine the impact of four major hormones on hepatocyte metabolism. Fold changes in the expression patterns of genes involved in the regulation of **(A)** Lipid oxidation **(B)** Mitochondrial metabolism and **(C)** Lipogenesis in the liver were determined. All the values are Means ± pooled SE of four independent replicate experiments/age. Each independent replicate experiment was performed on pooled hepatocytes isolated from 2–3 birds. Results are considered significant at *p* ≤ 0.05 following one way ANOVA. Different superscripts between bars in a panel indicates statistical significance from other treatments. CORT, Corticosterone; GH, Growth hormone; GCG, Glucagon; INS, Insulin; *PPARA*, Peroxisome proliferator activator receptor 1a; *CPT1A*, Carnitine palmitoyl transferase 1a; *LCAD*, Long-chain fatty acyl, *PGC1A*, Peroxisome proliferator-activated receptor gamma coactivator 1-alpha; *PCK1/2*, Phosphoenolpyruvate carboxykinase 1 and 2; *ACLY*, ATP citrate lyase; *FASN*, Fatty acid synthase; *SCD1*, Steroyl-CoA desaturase; *LSS*, Lanosterol synthase.

Isolated primary hepatocytes were incubated with four hormones whose levels are known to dynamically change during embryonic-to-neonatal development and in turn affect major metabolic events during development. The major changes in gene expression profiles in response to the hormonal treatments are highlighted below. The mRNA levels of *CPT1A* were lower (*p* ≤ 0.05) in the hepatocytes isolated from the neonatal livers treated with insulin (INS) when compared to the basal treatment (Figure 5A). Expression of the cytosolic Phosphoenolpyruvate carboxykinase (*PCK1*) was downregulated (*p* ≤ 0.05) in both the embryonic and neonatal hepatocytes in response to INS, compared to the basal treatment (Figure 5B). Glucagon treatment did not produce any significant effects on the mRNA profiles, compared to the basal treatment of the hepatocytes. Expression of mRNA for lipogenic genes *FASN* and *LSS* were significantly upregulated (*p* ≤ 0.05) upon INS treatment in the embryonic liver, compared to the basal treated groups (Figure 5C). In the neonatal hepatocytes, only *FASN* showed significant upregulation (*p* ≤ 0.05) upon INS treatment, compared to basal (Figure 5C). While the GCG induced changes in lipogenic gene expression of *FASN*, *SCD1* and *LSS* were lower than their corresponding expression in the embryonic hepatocytes treated with INS, the GCG induced changes in gene expression remained similar to basal treatment. Overall, these results suggest that insulin had the most prominent impact on hepatocyte lipid metabolism and lipogenesis in both the embryonic and neonatal stages.

### Impact of the Hormonal Stimuli on the Essential Amino Acid Content of Isolated Hepatocytes

**FIGURE 6:**
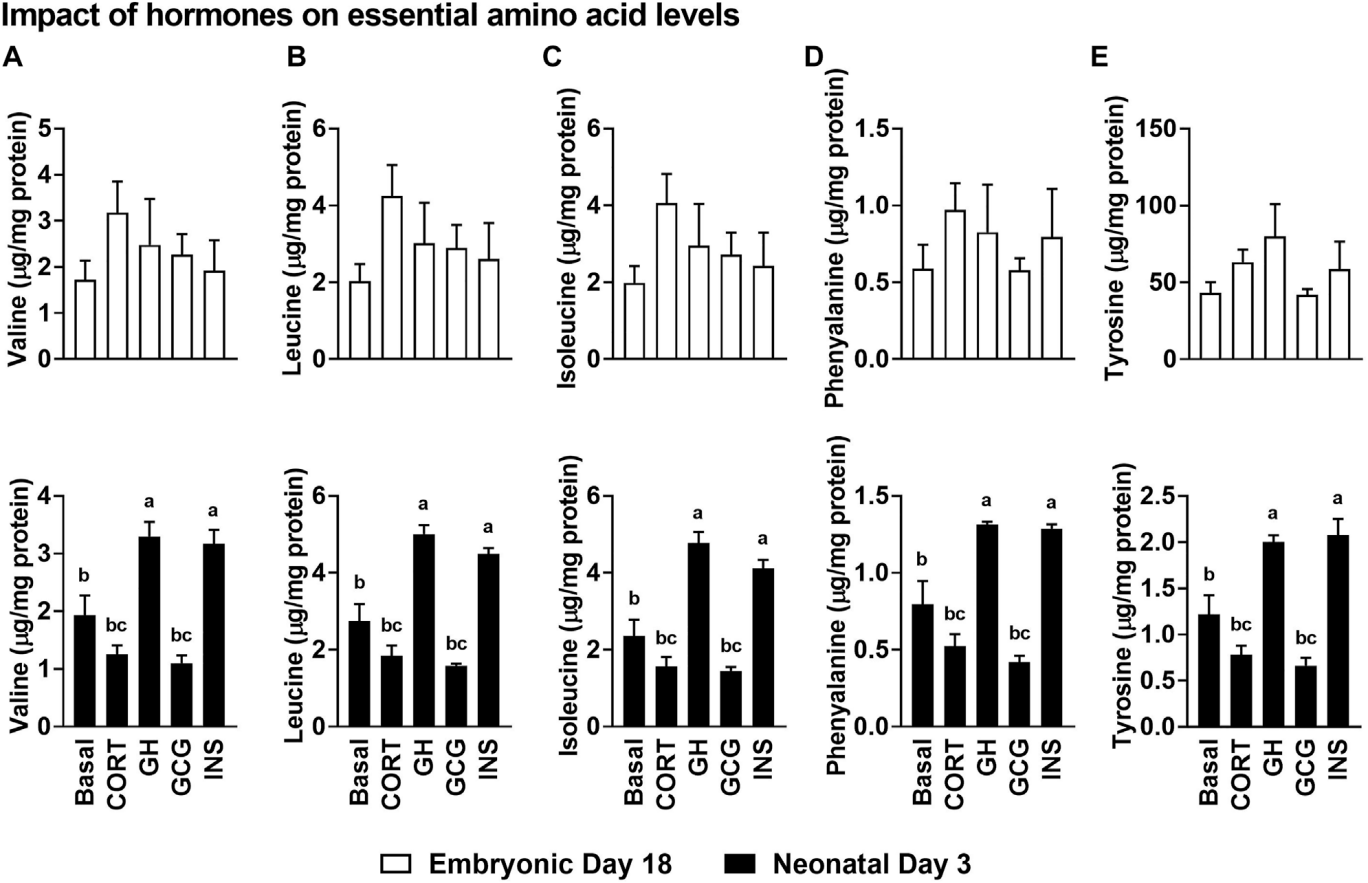
Impact of the hormonal stimuli on the essential amino acid content of isolated hepatocytes. Impact of hormones on levels of branched-chain amino acids, **(A)** Valine, **(B)** Leucine and **(C)** Isoleucine and the essential amino acids **(D)** Phenylalanine and **(E)** Tyrosine. All the values are Means ± SEM of four independent replicates/group. Each replicate consisted of pooled hepatocytes isolated from 2–3 birds. Results are considered significant at *p* ≤ 0.05 following one way ANOVA. Different superscripts between bars in a panel indicates statistical significance from other treatments. CORT, Corticosterone; GH, Growth hormone; GCG, Glucagon; INS, Insulin.

Hormonal treatments did not alter the levels of any of the essential amino acids in the hepatocytes isolated from embryonic day 18 liver (Figure 6). Interestingly, there was a significant increase in the levels of all the essential amino acids following growth hormone (GH) and INS treatments (*p* ≤ 0.05), compared to the basal treatment in neonatal hepatocytes. Contrary to the higher levels of all the essential amino acids in INS and GH treated hepatocytes, corticosterone (CORT) and GCG treatments resulted in lower levels of these amino acids in the neonatal hepatocytes (*p* ≤ 0.05). Considering the anabolic roles of INS and GH, these results could indicate the pivotal roles these essential amino acids are playing to support the active anabolic milieu in the neonatal hepatocyte, including rates of protein synthesis.

## Discussion

The embryonic-to-neonatal transition period in chicken provides a unique window to understand the metabolic mechanisms that help to promote rapid and healthy tissue development. Specifically in the liver, this window is unique because, the late term embryonic chicken liver (>day-16 of incubation) derives >90% of its energy through the oxidation of yolk lipids (Hazelwood, 1971; Noble and Cocchi, 1990; Moran Jr, 2007). Further, immediately after hatch, hepatic *de novo* lipogenesis is dramatically upregulated several hundred fold in response to the carbohydrate rich environment provided by the diet (Hicks et al., 2017; Surugihalli et al., 2019). This is interesting considering the fact that the lessons from mouse models and human subjects provide contrary evidence. In these species, a metabolic environment favoring high rates of lipid accumulation and high rates of lipid oxidation contributes to the etiology of fatty liver disease syndrome (Lambert et al., 2014; Patterson et al., 2016; Satapati et al., 2016; Kattapuram et al., 2021). However, during embryonic-to-neonatal transition in chicken, mitochondrial lipid oxidation is optimally coupled with *de novo* lipogenesis, in turn abating the onset and progression of hepatocellular stress (Surugihalli et al., 2019). Understanding mechanisms contributing to this synergy is of great significance to optimize embryonic-to-neonatal development and towards the management of fatty liver syndromes. This makes the embryonic-to-neonatal transition period in chicken is an attractive metabolic model to probe mechanisms regulating hepatocellular function, and thus warranting a detailed characterization of liver metabolism during this unique developmental period.

The embryonic-to-neonatal transition period is a period of active growth and metabolic development. With this in mind, we first profiled the circulating levels of amino acids and organic acid TCA cycle intermediates in embryos and neonatal chicken. The higher levels of circulating non-essential amino acids we observed in the neonatal chicken (e.g., alanine, aspartate and glutamate, Figure 1B) could point to their higher requirement as metabolic substrates to support the rapid growth of the hatchlings. There was also a parallel increase in the plasma organic acid TCA cycle intermediates (e.g., pyruvate, lactate, fumarate, α-ketoglutarate, malate and citrate, Figure 1C) in the neonatal circulation, many of which are carbon substrates for amino acid synthesis and transamination reactions. In the neonatal chicken liver, significant increases, relative to their embryonic counterparts, were only observed for aspartate, glutamate and serine. While pyruvate and lactate levels were higher in the neonatal liver, potentially a reflection of high rates of carbohydrate oxidation, the levels of other TCA cycle intermediates remained similar between groups. In spite of this, there was a more than 10-fold drop in circulating β-hydroxybutyrate levels from the embryonic-to-neonatal stage, clearly demonstrating the switch from free fatty acid utilization in the embryos to lipid synthesis in the neonates (Surugihalli et al., 2019). This metabolic switch was further substantiated by the higher rates of triglyceride accumulation along with the dramatic upregulation of lipogenic gene expression in the neonatal liver. The higher lipid accumulation in the neonatal liver occurred simultaneously with the lower expression profiles of genes involved in lipid oxidation. Liver is the primary lipogenic organ in the chicken (Zaefarian et al., 2019; Liu et al., 2020) and the metabolic milieu in the liver is primed to upregulate *de novo* lipogenesis immediately after hatch and on exposure to a carbohydrate rich dietary environment (Hicks et al., 2017; Surugihalli et al., 2019). The cytoplasmic network of *de novo* lipogenesis relies on and shares metabolic and molecular mediators of mitochondrial function, to upregulate its activity. In fact, the dramatic upregulation of *de novo* lipogenesis in the neonatal liver occurs simultaneously and in the presence of active TCA cycle metabolism (Surugihalli et al., 2019). Interestingly, such a metabolic milieu is also evident during NAFLD, which promote the onset of hepatocellular stress (Patterson et al., 2016; Satapati et al., 2016). The apparent absence of hepatocellular stress in the neonatal chicken liver points to the metabolic synergy between the mitochondrial oxidative networks, the cytoplasmic lipid synthesis machinery and the antioxidant defense systems during embryonic-to-neonatal transition (Surugihalli et al., 2019 ).

Isolated primary hepatocytes are widely utilized as an *in vitro* model system to investigate several facets of hepatic function, including lipid accumulation, mitochondrial function and insulin signaling in a variety of species (Nussler et al., 2001; Soldatow et al., 2013; Nagarajan et al., 2019). Based on this premise, we tested whether the primary hepatocytes isolated from the embryonic and the neonatal liver were able to reproduce the characteristics of the metabolic shift that we observed in the liver tissue. Indeed, the hepatocytes isolated from the neonatal liver had significantly higher triglyceride accumulation compared to their embryonic counterparts, as indicated by the significantly higher levels of fatty acid methyl esters in the nd3 hepatocytes. While the lactate content of the neonatal hepatocytes were significantly higher, the TCA cycle intermediates α-ketoglutarate and malate were significantly lower compared to the embryonic hepatocytes. Along with these differences in lipid accumulation and TCA cycle intermediates, the hepatocytes isolated from both the embryonic and neonatal liver elicited a robust response to insulin stimuli, indicated by the higher rates of AKT phosphorylation in both groups. More importantly, the AKT phosphorylation rates are significantly higher in the nd3 livers, illustrative of robust induction of insulin signaling, and further, coordinating the higher rates of lipid accumulation in these neonatal livers. Considering the significance of identifying mechanisms regulating mitochondrial function and its cross talk with cytoplasmic networks including lipogenesis, cultured primary hepatocytes could be a valuable model system.

We further tested the impact of the hormonal stimuli (insulin, glucagon, growth hormone and corticosterone) on the intermediary metabolism of cultured primary hepatocytes. It is well known that these hormones are major players in the remodeling of growth and intermediary metabolism during embryonic-to-neonatal development, with abundant expression of their respective receptors in the liver tissue (Hazelwood, 1971; Langslow et al., 1979; Jenkins and Porter, 2004; Lu et al., 2007; Wang et al., 2014). We tested the responsiveness of the primary hepatocytes to these hormones, by evaluating changes in expression of genes related to lipid oxidation and lipogenesis. While the response of several of the genes to these four hormonal stimuli presented a complex story, it revealed the major impact of insulin. Insulin stimuli resulted in lower mRNA levels of multiple genes involved in lipid oxidation, while increasing the mRNA levels of multiple genes involved in lipogenesis. The impact of glucagon on expression of genes involved in lipid oxidation and lipogenesis was not significantly different from that of the basal treatment. Overall, these results provided validation towards the utility of embryonic and neonatal derived primary hepatocytes as a potential metabolic model for probing changes in mitochondrial metabolism and lipogenesis, especially under insulin action.

The impact of these studies can be summarized towards addressing two major areas. 1) Outbreak of metabolic disorders like FLHS is one of the major causes of death in poultry flocks with over 5% mortality, especially during the laying cycle (Julian, 2005; Trott et al., 2013). Outbreak of hepatic lipidosis in turkey causes 0.7–17% mortality (Julian, 2005). Fatty liver and kidney disease (FLKS) is another known metabolic disorder that affects broiler chicken (2–3 weeks of age) and is associated with excess lipid deposition in kidney and liver and increased weight (Julian, 2005). Further, loss of chicken due to sudden deaths caused from FLHS/FLKS would affect the quality and quantity of meat and eggs produced (Trott et al., 2013). The etiology of these liver disorders warrants further investigations, and we believe the embryonic-to-neonatal transition period could hold clues to how the synergy between the intermediary metabolic networks in the liver can help prevent the negative outcomes associated with fatty liver syndromes. 2) Losses in productivity from high morbidity and mortality rates during the first week post-hatch is also a significant economic burden to the poultry industry (Noble and Cocchi, 1990; Wilson, 1991; Xin et al., 1994; Peebles et al., 2004). Avenues to optimize embryonic-to-neonatal transition, including the use of *in ovo* nutrient injection strategies (Kadam et al., 2013; Peebles, 2018; Neves et al., 2020), early post-hatch dietary modifications (Jha et al., 2019; Ravindran and Abdollahi, 2021) etc., are being investigated to optimize this critical transition of the embryo to the neonate. In summary, isolated hepatocytes from embryonic and neonatal chicken can be a reliable metabolic model to investigate the optimal remodeling of mitochondrial metabolism and its synergy to accommodate rapid rates of lipogenesis, while avoiding mechanisms promoting hepatocellular stress.

## Data Availability

The raw data supporting the conclusion of this article will be made available by the authors, without undue reservation.
