# Supplementary material for: Remodeling of Hepatocyte Mitochondrial Metabolism and De Novo Lipogenesis During the Embryonic-to-Neonatal Transition in Chickens
Source: Front Physiol. 2022 Apr 21;13:870451. doi: 10.3389/fphys.2022.870451 (PMC9068877; doi:10.3389/fphys.2022.870451)
Supplement: Supplementary file 1 [file Table1.pdf]

Supplementary Table 1: List of primers utilized for quantitative PCR

Gene	Forward Primer	Reverse Primer
PPARA	CAATGCACTGGAACTGGATG	ACATGCACAATGCTCTCCTG
CPT1A	GGGTGGCTAGGTTTGGATAA	CAGCCTCTAAATGCACCAAA
LCAD	GGGAGAGCACCAGGAAGTTCT	GCCATCCTTCTCCCATTCTG
PCG1A	CATGTGCAACCAGGACTCTGT	GATGTTGGCAGGCTCATTGC
PCK1	GCAGGGGTTATGATGAGAAGT	ACGGATCACAGTTTTGAAGAC
PCK2	CCTTCGCCATGAGCCCCTTTTTC	CAGCTCCGCCATGACATCCCT
ACLY	CTTTTAAGGGCATTGTTAGAGCAAT	CCTCACCTCGTGCTCTTTCAG
FASN	TTCTGATTCTGGCTCCACTG	CCTGCTTAGCACTCTCAACG
SCD1	CTGCTCACATGTTTGGCAAT	TGGAGTAGTCGTAGGGGAATG
LSS	GCACGTATGTGCAGAGTTCC	GGCAGCTGCTTATCAATCAA
GAPDH	GATTTAATGAGCCATTCGCAGTT	CCCAGCGTGCATGTCTAAGTAC
